# Recycling Textiles: From Post-Consumer Polyester Garments to Materials for Injection Molding

**DOI:** 10.3390/polym17060748

**Published:** 2025-03-12

**Authors:** Sabrina Bianchi, Michele Pinna, Flavia Bartoli, Pierpaolo Minei, Daniele Filidei, Maria-Beatrice Coltelli

**Affiliations:** 1 SPIN-PET S.r.l., Viale R. Piaggio 32, 56025 Pontedera, Italy; bianchi@spinpet.it (S.B.); pinna@spinpet.it (M.P.); bartoli@spinpet.it (F.B.); minei@spinpet.it (P.M.); filidei@spinpet.it (D.F.); 2 Department of Civil and Industrial Engineering, University of Pisa, 56122 Pisa, Italy

**Keywords:** recycling, chain extender, polyester, circular, textile, garments, compaction, monomaterial

## Abstract

The significant waste generated by the fashion industry necessitates sustainable textile recycling strategies. Polyester, made from poly(ethylene terephthalate) (PET), is abundant in post-consumer textiles. Technologies have been developed to convert low-density garment waste into flakes, but the role of color sorting in achieving uniform aesthetics in injection-moldable plastics remains underexplored. This study compares materials extruded from dark color-sorted polyester garment flakes with those from light-color flakes in terms of processability in extrusion and injection molding. The properties examined include melt fluidity, injection molding shrinkage, and mechanical and thermal properties. Commercial chain extenders with anhydride, oxazoline, or epoxide reactive groups were added during extrusion. Interestingly, only dark-colored extruded pellets showed significant degradation, but all the chain extenders allowed melt fluidity to be controlled during reprocessing. The bisoxazoline-based additive was the most promising, due to the highly improved ductility of the samples, regardless of whether they were dark-colored or light-colored. The results indicate significant potential for the industrial recycling of post-consumer textiles and highlight the industrial feasibility of repurposing post-consumer polyester garments. This approach not only supports initiatives of circular economy but also offers a viable solution for managing textile waste, particularly in the fashion industry. Additionally, the suggested recycling route combats the production of microplastics.

## 1. Introduction

The textile industry is a significant contributor to global waste and environmental issues. The textile industry generates approximately 58 million tons of plastic waste annually, making it the third-largest contributor after packaging and construction. The industry is also responsible for 20% of global wastewater and 10% of global carbon emissions [1,2,3]. The impact on biodiversity is equally alarming: 33% of the world’s insecticides are used in fabric production. Despite these staggering figures, the response to recycling is minimal. Only a mere 1% of global clothes are recycled, while a whopping 87% end up being incinerated or in landfills. This is mainly due to the limited development of recycling infrastructure in the industry.

Despite the abovementioned issues, the last two decades have witnessed a dramatic surge in global textile fiber production. Back in 2000, production stood at 58 million tonnes. Fast forward to 2020, and this figure has almost doubled, reaching an impressive 109 million tonnes. The trend shows no signs of slowing down, with projections suggesting that production will soar to 145 million tonnes by 2030 [4]. The implications of this growth are profound, affecting not just the environment but also the sustainability practices within the industry [5].

Textile fibers in commercial products are generally classified as either natural or man-made. At the molecular level, both types consist of polymer chains, with each fiber type having its own specific monomers and polymer linkages.

Man-made fibers are produced by extruding molten or dissolved polymer through small openings in a spinneret. This process can yield filament yarns, in which the yarn consists of a single continuous fiber, or spun yarns, where shorter fiber segments are twisted together. Common synthetic polymers used in textile products include polyester, polyacrylic, polyamide, elastane, and polypropylene. Among these, polyester is the most widely used due to its durability and relatively low cost compared to other fibers [6]. Polyester, known as poly(ethylene terephthalate) (PET), is also commonly used for producing packaging containers, such as bottles.

The polyester used in textiles has a lower molecular weight and higher crystallinity than the PET used in bottles. Historically, PET for textiles was produced through the polycondensation of dimethyl terephthalate and ethylene glycol, while PET for bottles was made from terephthalic acid and ethylene glycol. The latter method leads to less regular polymer chains, making it more suitable for creating an amorphous material. Recently, however, the synthesis using terephthalic acid has become the dominant method due to its economic and industrial advantages [7]. In the last decades, PET from post-consumer bottles was also used for producing fibers and textiles [8,9], but European laws are promoting the use of recycled PET in food packaging too [10], so that in the future this source can be less available. This perspective can make the use of post-consumer PET from other sources, for instance from textile itself, more attractive. Nevertheless, its different macromolecular structure as well as its higher crystallinity could require a specific approach in designing the recycling methodologies.

In textile products, PET fibers are often used alone or blended with natural fibers such as cotton to improve breathability, or with elastane to create stretch fabrics.

Synthetic microfibers are a major type of microplastics polluting marine and freshwater environments, primarily originating from textile industry discharges, domestic and industrial effluents, illegal dumping, and abandoned fishing gear [11,12]. Textile PET microfibers are the most common microplastics found in wastewater treatment plants [13,14,15]. Recycling fibrous PET garments into thicker plastic items through a circular approach can significantly benefit the environment. This process helps reduce the amount of microfibers released into water bodies, minimizes waste, and promotes the reuse of materials, contributing to a more sustainable and eco-friendly system.

The recycling of PET-based materials from textiles can be achieved by melting the polymers above their melting point and reprocessing them. However, several challenges need to be addressed:

1. Material Sorting: It is essential to sort homogeneous materials (monomaterials). In fact, when PET is mixed with cotton or other synthetic polymers, polymer composites or blends are formed in reprocessing [16]. Due to the different chemical structures, the compatibility between the polymers is limited, resulting in poor mechanical properties [17]. In the past several reactive processing methodologies were developed by researchers to overcome this issue in PET blends recycling [18,19,20,21]. Nowadays, sorting techniques based on spectroscopy [22], like for instance near-infrared spectroscopy [23,24] coupled with artificial intelligence systems [25], can be applied on an industrial scale.
2. Compaction: Increasing the density of textile PET material through proper compaction systems is necessary, as suggested by several researchers [26,27,28]. Typically, a combination of increased temperature and pressure followed by grinding can transform the selected textiles into flakes [29].
3. Compounding: The material obtained must be compounded by extrusion with appropriate additives, depending on the final application. This step could allow the flakes to be transformed into pellets suitable for injection molding, compression molding, or additive manufacturing. However, the material thus obtained should show the desired rheological properties. Generally, PET undergoes chain scission during compounding operations, resulting in a material with very high fluidity, not suitable to be processed in manufacturing technologies where a significant melt strength is required.

Additionally, the ability to sort polyester textiles by color could enable companies to select materials with specific aesthetic properties [30] for applications, especially in design-focused sectors.

An important feature of post-consumer PET is its melt fluidity. In fact, its reprocessing is highly influenced by it. For instance, for injection molding applications, a higher melt fluidity than for sheet casting is preferable. However, the repeated processing of PET generally induces a progressive decrease in its molecular weight and a consequent increase in melt fluidity [31]. This can lead to a material that cannot undergo further reprocessing steps. This effect is attributable to the chain scission due to water vapor or humidity. In fact, water molecules can react with the ester groups of the polyester chain inducing chain scission. For controlling this effect, during reprocessing operations applied to polyesters, chain extenders are often used for modulating melt fluidity [32,33,34,35]. These additives consist of bifunctional or multifunctional molecules bearing chemical groups that are reactive towards the polyesters’ terminal groups (hydroxyl or carboxylic groups). The reactivity of these groups should be very high and their reaction fast enough to be compatible with the processing operations, often lasting less than 1 min. The impact of adopted methodologies in the reactive processing of polyesters is also nowadays under debate [36]. Regarding functional groups, epoxide [37,38,39,40], oxazoline [41,42], anhydride [43,44], or isocyanate [45,46] groups were mainly considered because they respect the abovementioned requisites. In particular, epoxide is more reactive towards the carboxylic groups of polyesters, but it can partially react also with hydroxyl ones [46]; oxazoline [47] reacts with carboxylic groups [46] and anhydride [43] reacts mainly with hydroxyl terminal groups. Isocyanate group reacts with both hydroxyl and carboxylic groups [41]; recently, in the synthesis of PET and polyethylene furanoate (PEF) copolymers with isosorbide, diguaiacyl oxalate (DGO) [48] was used as a chain extender, with the advantage of not altering the chains’ structure and being used in low concentrations. However, this chain extender, inducing the emission of ethyl oxalate, could not be suitable for reactive processing, and its commercial availability is still limited.

The macromolecular design of the obtained polyester is also strongly influenced by the molecular weight and structure of the chain extender. In fact, the chain extender can bear two reactive groups, inducing the maintenance of a linear structure, or three and more groups, inducing branching. When oligomers bearing several groups, like Joncryl products, consisting of a terpolymer including epoxide functionalities, were used, highly branched structures were obtained [39,49]. While PET modified with bifunctional chain extenders resulted in linear macromolecular chains, the Joncryl-modified PET showed a strongly branched molecular structure [50]. The flexibility of the chain extender also influences the final properties of PET, especially the rheological [51] and thermal ones [52]. Similar investigations, comparing different commercial chain extenders, were never carried out using PET coming from post-consumer garments.

In this paper, after evaluating the potential of sorting post-consumer polyester apparel by color, PET textile garments (sorted into dark and light-colored) were transformed into flakes through compaction. These flakes were then extruded with bifunctional or polyfunctional chain extenders containing anhydride, oxazoline, or epoxide groups. The melt fluidity, as well as shrinkage and thermo-mechanical properties of the injection-molded specimens were analyzed to determine the effectiveness of these methodologies for recycling PET from textile garments.

## 2. Materials and Methods

### 2.1. Materials

**Figure 1:**
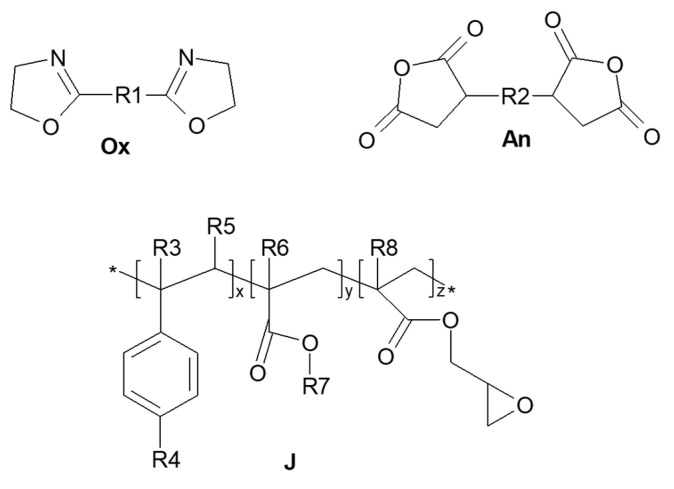
Structure of the bisoxazoline, dianhydride, and general structure of the styrene-acrylic multifunctional oligomeric chain extenders. * R3–R7 are H, CH3, a higher alkyl group or combinations of them; R8 is an alkyl group. x, y and z are between 1 and 20 [53].

Materials used in this study were 100% polyester post-consumer garments. The used chain extenders were selected considering their wide availability in the market, affordable price, and effectiveness due to their high reactivity in polycondensation polymer compounds. They were Nexamite^®^ M021200 (An) from Nexam Chemical Holding AB (Lomma, Sweden) consisting of a polyester based masterbatch containing 12% by weight of a dianhydride; NEXAMITE^®^ M992000 (Ox) from Nexam Chemical Holding AB (Lomma, Sweden), consisting of a polyester based masterbatch containing 20% by weight of a bisoxazoline; and Joncryl 4468 (J) from BASF (Ludwigshafen, Germany), consisting of a terpolymer containing epoxydic reactive groups (Figure 1). Joncryl ADR 4468, BASF (Ludwigshafen, Germany) is an epoxy oligomer. It is an oligomeric chain extender, carrying about 23 average epoxy groups per molecule (epoxy equivalent weight: 310 g/mol). Its molecular weight is 7250 g/mol, the density is 1.08 g/cm^3^, and it appears as solid flakes.

**Figure 2:**
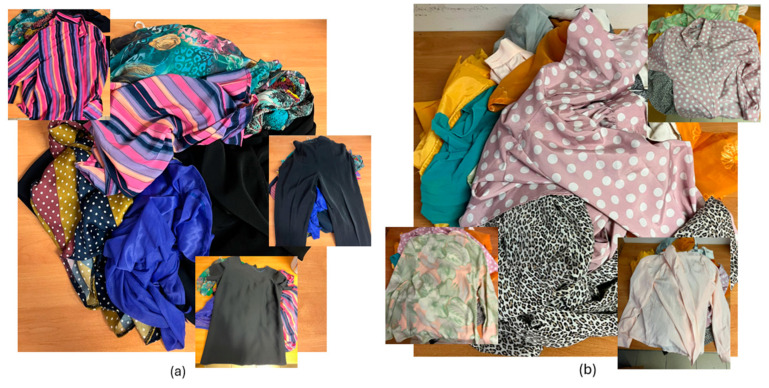
100% polyester post-consumer garments. (**a**) Dark colored selection; (**b**) Light colored selection.

Used and discarded garments were purchased from Mani Tese ONG (Milan, Italy), section of Florence, among those remaining after the selection of garments suitable for sale as vintage or second-hand clothing. Four bags of approximately 120 L of such garments were purchased and manually sorted, based on their tags, to identify those made of 100% polyester. From this selection (after a preliminary work on flakes obtained by white, yellow, red, blue and black garments, suggesting this methodology) a further subdivision was made between light-colored and dark-colored garments (Figure 2). All rigid components (zippers, buttons, etc.) and any materials visibly different from polyester (adhesive linings, decals, etc.) were manually removed from the garments (less than 5% by weight).

### 2.2. Processing

**Figure 3:**
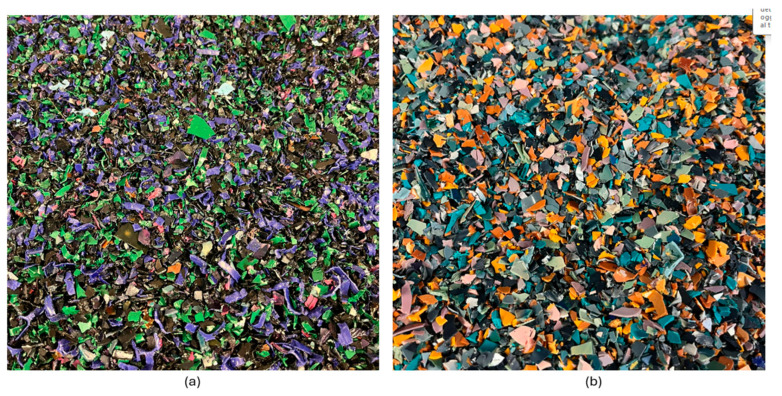
100% polyester flakes from compaction of post-consumer garments. (**a**) Dark colored selection; (**b**) Light colored selection.

The garments thus selected and prepared were compacted and reduced into rigid flakes (Figure 3) using the patented process owned by Spin-PET srl [29].

The flakes were dried in a ventilated oven at 150 °C for 3 h before being processed to prevent hydrolytic degradation during processing. The reactive processing was performed in a single-screw extruder (Brabender Measuring Extruder 19/25 D connected to Plastograph Can from Brabender GmbH, Duisburg, Germany) equipped with a compounding screw (Barrier Screw (Maillefer-type), compression ratio: 2.5:1). The rotor speed was set to 50 rpm, and the following temperature profile was applied: 250, 260, 260, and 250 °C. The compounds were prepared with compositions reported in Table 1. Compositions were selected considering the data available on products’ technical sheets and our previous knowledge regarding the use of Joncryl products in polyesters [46,54,55].

Specimens were prepared with a Micro-Jet pneumatic Rondol (Rondol Technology Ltd, Nancy, France) press at 260 °C using pre-dried (3 h at 150 °C) pellets and with piston speed 80 mm/s maintaining the mold at room temperature. All specimens were quenched by immersion in water at 0 °C. The different processing steps are summarized in Table 2.

### 2.3. Characterization

The compounds of PET obtained from recycled garments (rG-PET) were characterized in terms of rheological, mechanical, and thermal properties. The melt flow rate (MFR), expressed as g of material per 10 min, was determined using a CEAST PIN 7026 melt flow module equipped with “VisualMELT 2.0” software (CEAST, Torino, Italy) which provides melt volume rate (MVR) data. The melt flow rate was measured at 260 °C with an overhead weight of 2.16 kg (ASTM D1238 [56]). The samples were kept for 3 h in a 150 °C pre-heated oven before the MFR measurement. The tensile and the flexural mechanical properties were analyzed with a Shimadzu AutoGraph Serie AGS-X dynamometer equipped with a 5 kN load cell (Shimadzu, Kyoto, Japan). For tensile measurements, the test specimens were prepared according to ISO-527-2 [57] from an injection-molded dumbbell sample (1BA Type) and analyzed at room temperature with a stretch rate of 1 mm/min. Each data is the average of at least five tested specimens.

For flexural measurements, specimens with dimensions of 80 × 10 × 4 mm have been prepared as required by ISO 178 standards [58].

DSC thermograms were recorded on a PYRIS Diamond Perkin Elmer calorimeter (Perkin Elmer, Waltham, MA, USA). All samples were heated at a rate of 10 °C/min from 25 °C to 270 °C and held at this temperature for 3 min to eliminate thermal history. The samples were then cooled to 25 °C at a rate of 20 °C/min, held for 3 min, and subsequently reheated to 270 °C at a rate of 10 °C/min. The crystallization temperature (Tc), crystallization enthalpy (ΔHc), melting temperature (Tm), and melting enthalpy (ΔHm) were obtained from the cooling scan and the second heating stage, respectively.

The crystallinity (X) of PET was calculated using Equation (1):

(1) $$X=\frac{∆Hm}{∆Hmi}\times 100$$

where ΔHmi represents the melting enthalpy for 100% crystallinity of PET (140 J/g) [59].

## 3. Results

Using the compaction patented method, flakes from garments with dimensions and apparent density suitable for direct extrusion were produced. Flakes were obtained without any pretreatment of the garments themselves, apart from the removal of non-meltable components (e.g., zippers, buttons). A preliminary study of white, yellow, red, blue and black selected garments was carried out considering the MFR and MVR of the respective compacted flakes (Table 3). It could be observed that the value of MFR and MVR showed a general increasing trend going from lighter to darker colors. These results suggest that it is reasonable to select garments mainly in groups of light and dark colors. On the other hand, most post-consumer garments are in patterned colors, so color-by-color selection is necessarily approximate.

After the selection and compaction of light-colored and dark-colored garments, the MFR of the obtained flakes was measured: that of dark colored flakes was 21.3 and that of light-colored flakes was 24.8.

To enhance the mechanical and rheological properties of extruded rG-PET for upcycling into higher-value applications, various commercially available reactive modifiers were tested as melt viscosity regulators. The MFR (Table 4) and the mechanical properties of the modified rG-PET were used as key parameters to evaluate and compare their reactivity.

**Figure 4:**
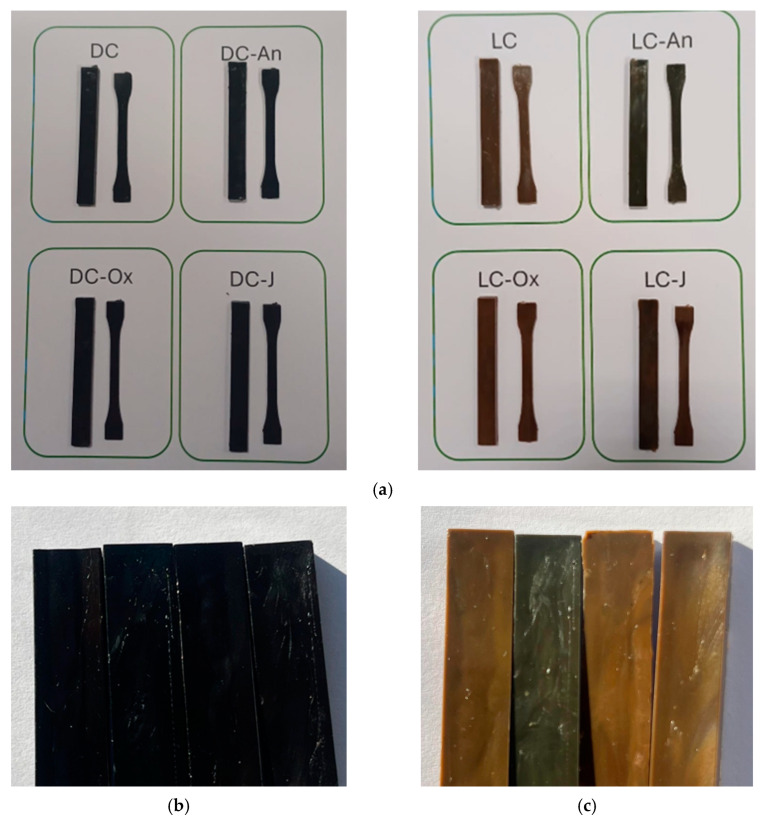
(**a**) Injection molded specimens for flexural and tensile properties measurements; (**b**) dark-color specimens: from left to right DC; DC-An; DC-OX; DC-J; (**c**) light-color specimens: from left to right LC; LC-An; LC-OX; LC-J.

The extrusion of all compounds allowed for the recovery of a continuous and homogeneous filament, which was pelletized after cooling in water at room temperature. The resulting pellets were then dried and used for the preparation of specimens via injection molding (Figure 4). MFRs higher than 10 g/10 min at the processing temperature are generally required for having a good processability by injection molding [60]. All the tested materials fulfilled this requirement, except for LC-J, which showed lower fluidity. However, it was possible to obtain specimens from LC-J without specific modifications of the injection molding parameters.

In the light-colored specimens, it is noticeable that the use of the chain extender based on pyromellitic anhydride slightly modified the color of the compound compared to both the unmodified compound and those containing “non-acidic” chain extenders. It can be hypothesized that this effect is due to the interaction of the dyes, or part of them, with the acidity developed by the opening of the anhydride functionality in the LC-An compound. The same interaction with a portion of the dyes may have also occurred in the DC-An compound; however, it might not be as evident due to the overall darker color.

Mechanical properties are key indicators of a material’s performance and are essential for evaluating its suitability for practical applications. In Table 5 the stress at yield, the Young’s modulus, and the deformation at break results for rG-PET compounds in the presence of chain extenders are reported.

**Figure 5:**
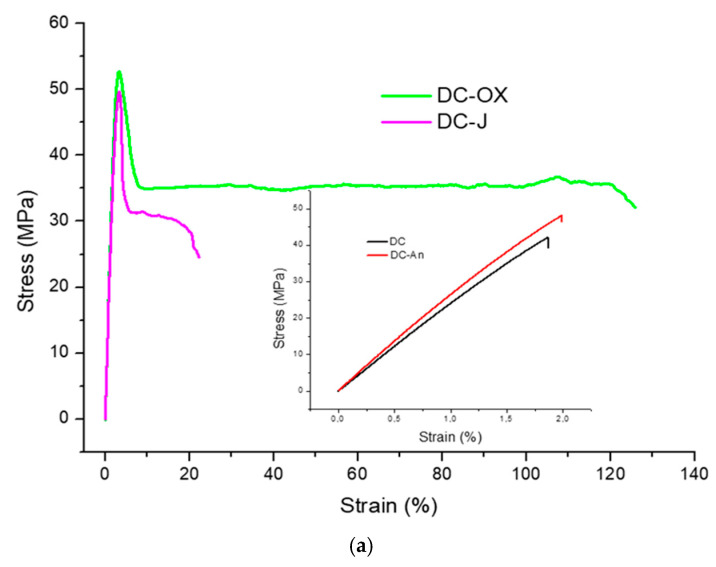

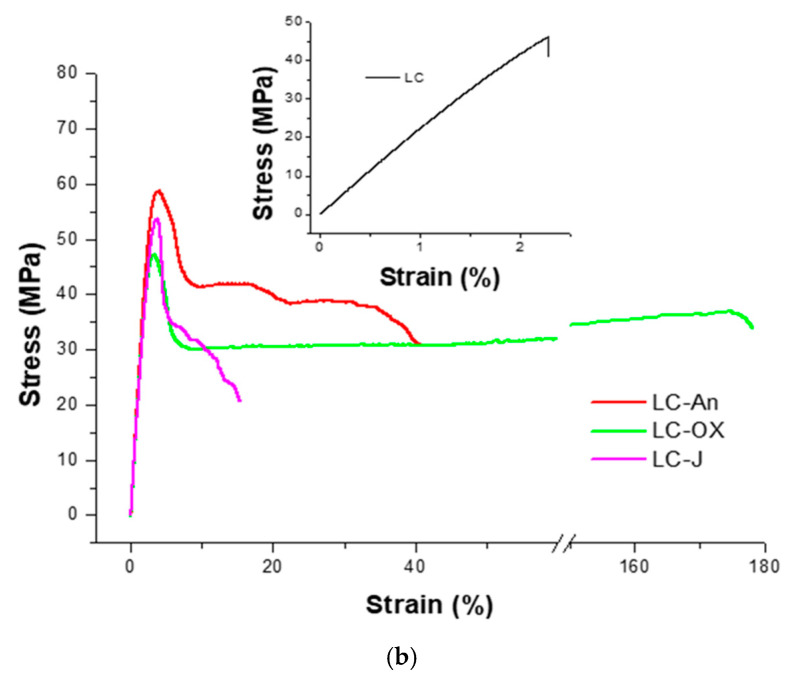
Representative stress-strain curves comparison: (**a**) between DC-Ox and DC-J; (**b**) between LC-An, LC-Ox, and LC-J. DC-Ox and LC-OX curves suggest a high ductility of these compounds.

Generally speaking, the addition of chain extenders enhanced the mechanical properties of rG-PET. The Young’s modulus exhibited no substantial variations, maintaining values that are only slightly lower than those of a recycled bottle-grade PET [61]. The elongation at break varied significantly depending on the chain extender used. Samples obtained from flakes without the addition of any chain extender exhibited brittle failure, breaking after minimal deformation (approximately 2% elongation). The incorporation of chain extenders led to a marked increase in elongation at break, up to 171% in the case of light-colored sample and oxazoline-based chain extender (Figure 5). The use of chain extenders, with the sole exception of the DC-An compound, enables plastic deformation and the appearance of a yield point in the stress-strain curve. The yield stress, which defines the mechanical limit beyond which a material undergoes permanent deformation, is between 50 and 60 MPa. These values match those of a general-purpose injection-molding-grade PET [51]. In both DC and LC samples, the most effective chain extender is the one with oxazoline functionality.

Flexural modulus and other flexural properties for rG-PET compounds in the presence of chain extenders are reported in Table 6. The trends were very similar to those obtained by tensile tests. Also in this case, DC-Ox and LC-Ox showed the highest stress at break and deformation at break, in agreement with their improved ductility.

The shrinkage percentage in injection molding is a critical parameter that directly impacts the dimensional accuracy and overall quality of the final product. Accurate prediction and control of shrinkage are essential to minimize defects such as warping, sink marks, or dimensional inconsistencies [61,62]. Shrinkage is influenced by factors such as material properties, mold design, cooling rates, and processing conditions [62,63,64,65]. Properly managing this parameter ensures that the molded part meets design specifications, reduces the need for post-processing adjustments, and enhances production efficiency. In this study, under identical injection molding parameters, variations in the shrinkage percentage are primarily attributable to the type of chain extender used. The chain extender used influences the molecular structure and rheological behavior of the material, which in turn affects its shrinkage characteristics during the cooling phase. In Table 7 the shrinkage percentage is reported relatively to the different compounds.

The shrinkage behavior is generally associated to the thermal behavior of the material [66,67,68]. We can assume that the shrinkage in injection molding is due to the crystallization of the material, as crystalline regions have a higher density than amorphous ones. Higher shrinkage was observed in all samples in the transverse direction [69]. In fact, PET macromolecules are oriented in the flow direction and consequently the preferential formation of crystals occurs in the perpendicular direction [70]. Viora et al. [71], evidenced that recycled PET crystallizes faster and more easily than virgin PET. Moreover, they found that its quenching ability decreases. Although the injection-molded specimens were cooled maintaining the mold at room temperature and quenched at 0 °C, they could partially crystallize, determining the observed shrinkage values. Regarding DSC results (Table 8), in the thermograms of the second heating there was no crystallization during heating. Then, in good agreement with Viora et al.’s observations [71], all the samples crystallize completely during the cooling process.

LC samples have higher melting peak temperatures than DC, and this can be probably associated to their higher molecular weight, as shown by the MFR results (Table 4) [71,72,73,74]. The use of chain extenders, although inducing an increase in molecular weight, slightly decreases the temperature of the melting peak, perhaps because the introduced structural disorder increases the presence of lower-melting irregular crystals. It can be additionally concluded that the chain extenders used, in the selected concentrations, do not induce substantial changes in the thermal properties of the polyester. Crystallinity does not undergo significant alteration, although a slight increase is generally observed by using chain extenders. Thus, the shrinkage results (Table 7), although they concern molded pieces, seem to be in good agreement with the thermal behavior of the materials.

## 4. Discussion

The compaction patented method employed in this study does not require prior grinding, pulverization, shredding, or drying to prepare flakes. These flakes have a morphology and density that enable direct feeding into extruders, even into a single-screw extruder, thus ensuring continuous operation even under ‘full-feed’ conditions. The measured bulk densities of 580 g/L for the dark-colored selection and 575 g/L for the light-colored selection were obtained. These densities are similar or even higher than those of flakes from bottles or other rigid containers [75].

The MFR data are directly related to melt viscosity and therefore to molecular weight of the polymer, although significant deviations may occur as a result of branching.

Considering that the MFR of virgin fiber grade PET pellet is generally between 20 and 25 g/10 min [76] and that of DC and LC flakes (all measured at 260 °C with a weight of 2.16 kg) are very similar, it is evident that during the compaction process, the material does not undergo a significant degradation.

It is possible to observe that the MFR of the extruded rG-PET dark-colored samples was consistently higher than that of the light-colored samples (Table 4). It can also be hypothesized that this difference is related to the variation in coloration between the selections. In fact, polyester fibers are dyed with disperse dyes, which are non-ionic, water-insoluble colorants with a different chemical nature [76,77,78]. For example, anthraquinone-based dyes are used especially for blue and red shades (e.g., Disperse Blue 56, Disperse Red 60) and have generally high thermal stability, whereas azo-based dyes, widely used for yellow, orange, and black (e.g., Disperse Yellow 23, Disperse Black 9), have lower thermal stability. The typical dye concentration in mass coloration ranges from 0.01% to 2% of the polymer weight, depending on the desired shade intensity. Light shades generally require concentrations between 0.01% and 0.1%, while deeper and more intense colors necessitate 0.5% to 2%. Therefore, a reasonable hypothesis for the higher MFR observed in pellets from dark-colored garment selections compared to lighter-colored selections is the potential thermal degradation of colorants and, in particular, of Disperse Black 9, which is likely used more frequently and in higher concentrations in darker textiles. As evidenced systematically by Nguyen et al. [79], the degradation temperature (inflection points) of several azo-dyes is in the 200–270 °C range. Thus, during PET processing at high temperatures (~260 °C), the azo bond (-N=N-) in Disperse Black 9 may undergo cleavage, forming aromatic amines and nitrogen oxides (NOx) [80,81,82]. These degradation by-products could catalyze chain scission reactions in PET (reasonably due to the nucleophilic attack of amines), leading to a reduction in molecular weight and an increase in MFR. In contrast, lighter-colored garments, which generally contain lower concentrations of thermally sensitive azo dyes, might experience less degradation-induced chain scission, resulting in a comparatively lower MFR. In agreement with the proposed hypothesis, Mu et al. [83] have developed a method to separate polyester obtained from textiles from its azo dyes to allow a more effective recycling of the polymer. However, the degradation paths of polyesters correlated to the presence of azo dyes are still underexplored, and further research is required to confirm this hypothesis. In the case it will be confirmed, this could suggest the textile industry to replace azo dyes with more thermally stable dyes, not undergoing degradation during the recycling operations.

It is also notable that the patented fabric compaction process does not produce significant differences between the two selections, as deduced from MFR values of the flakes, further supporting the fact that this procedure enables the production of material suitable for standard extruders without significantly degrading its properties [29]. This suggests that this procedure for compaction effectively processes textile waste into a feedstock without reducing properties.

Regarding the decrease in MFR, the Joncryl chain extender was more efficient than the others, as evident by considering the difference in the MFR obtained with respect to the reference materials DC and LC (Table 9).

**Figure 6:**
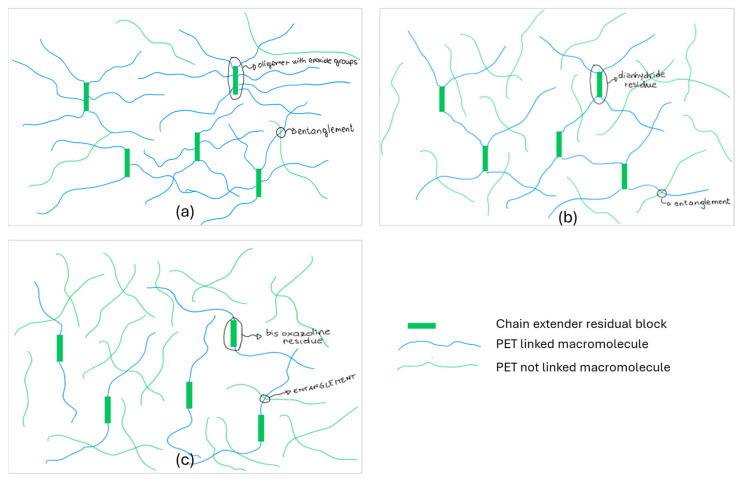
Possible macromolecular designs for (**a**) Joncryl (J); (**b**) dianhydride (An); (**c**) bisoxazoline (Ox) chain-extended PET (30 macromolecules were represented in each scheme). Chain extender residues and entanglements are represented. Due to the different number of links formed per molecule, the number of PET linked macromolecules (blue line) is in the order: J > An > Ox.

In fact, Joncryl has a higher number of reactive groups per molecule, thus its reactivity could generate branched structures dispersed in the PET matrix (Figure 6a). Regarding dianhydride, this chain extender was reported to produce up to four linkages per molecule [51]. For this reason, it resulted in a ΔMFR higher than that observed for bisoxazoline (Figure 6b). The bisoxazoline chain extender generated up to two linkages, and was the less efficient in increasing the molecular weight as it involved a lower number of PET macromolecules (Figure 6c), but, differently from the others, it left unchanged their linear feature.

The differences in macromolecular design affected the final mechanical properties. In fact, chain-extended materials showed an improved ductility, especially D-Ox and L-Ox samples (Table 8). This peculiar improvement correlates to the maintenance of a fully linear structure. With the other chain extenders, producing more than two linkages per molecule, the inter-macromolecular linkages involved more macromolecules, favoring the generation of net-shaped molecules, and this resulted in a material with an increased rigidity with respect to those chain extended with bisoxazoline, hence with a lower ductility.

The general differences between chain extenders are summarized in Table 10. Differences in tensile strength and modulus, and in shrinkage as well as in flexural modulus, were not significant. Tensile strengths were improved with respect to compounds without chain extenders and the obtained values were higher than 45 MPa.

## 5. Conclusions

After preliminary work indicating the inopportunity of selecting garments color-by-color for their up-cycling, light-colored and dark-colored garments were selected and compacted using a patented method to obtain flakes. These flakes were then extruded with or without chain extenders based on a dianhydride, a bisoxazoline, or a terpolymer bearing several epoxide groups per molecule.

The dark-colored material extruded without chain extenders exhibited higher fluidity (indicating a lower molecular weight) compared to the light-colored material. This result was tentatively explained by considering possible chain scission degradation pathways due to the presence of azo dyes. However, further research is necessary to clarify this significant point.

The chain extenders proved effective in regulating the melt fluidity, with Joncryl showing the highest decrease in melt flow rate. Dianhydride demonstrated a greater capacity to reduce melt fluidity compared to bisoxazoline. This was attributed to the fact that dianhydride can generate up to four linkages per molecule, whereas bisoxazoline can generate up to two.

All the tested chain extenders were effective in improving the ductility of the samples. Nevertheless, bisoxazoline showed the best results, which was attributed to the maintenance of a linear macromolecular structure. All the samples could be injection-molded, and their shrinkage was similar, being higher in the transverse direction compared to the flow direction. These results can be attributed to the crystallization of PET, which was only slightly affected by the addition of chain extenders.

This work demonstrated that polyester from post-consumer garments can be recycled through injection molding, and chain extension can be a convenient strategy, especially for dark polyester post-consumer samples. Moreover, understanding the different chemical behaviors of the chain extenders and the relationships between structure and final properties represents important know-how. This knowledge could suggest other recycling possibilities for the future, further supporting initiatives of circular economy. Future research deepening, such as testing additional additives or scaling up the process, could further strengthen the impact of this study.

Injection molding is a very widespread technology and suitable for mass production, such as objects, gadgets, packaging, jars, caps, household appliances cases, handles, etc. Therefore, the results obtained can suggest methodologies for large-scale recycling. This technology can be leveraged for large-scale recycling, but it requires a substantial supply of post-consumer polyester garments. Therefore, better organization and management of garment collection, along with necessary policy and logistic measures, are crucial.

An important aspect is linked to the fact that post-consumer PET products made up of fibers, therefore subject to the release of microplastics, are being replaced with products of higher thickness and therefore less harmful to the environment from this point of view.

## Figures and Tables

**Table 1:** Compounds composition.

Compound	PET Flakes (g)	Chain Extender (g)
DC	Dark Colored (200)	//
DC-An	Dark Colored (200)	Nexamite^®^ M021200 (6)
DC-Ox	Dark Colored (200)	Nexamite^®^ M992000 (6)
DC-J	Dark Colored (200)	Joncryl 4468 (1)
LC	Light Colored (200)	//
LC-An	Light Colored (200)	Nexamite^®^ M021200 (6)
LC-Ox	Light Colored (200)	Nexamite^®^ M992000 (6)
LC-J	Light Colored (200)	Joncryl 4468 (1)

**Table 2:** Summary of the processing steps for post-consumer polyester garments.

Processing Steps for Polyester Garments	Procedures and Parameters	Final Items
Compaction	Heating at 150–310 °C and grinding [29]	Flakes
Extrusion	Drying in a ventilated oven at 150 °C for 3 h; single-screw extrusion with rotor speed set to 50 rpm, and temperature profile: 250, 260, 260, and 250 °C	Granules (pellets)
Injection molding	Drying in a ventilated oven at 150 °C for 3 h; setting 260 °C and piston speed 80 mm/s; mold at room temperature; specimens quenched by immersion in water at 0 °C.	Specimens

**Table 3:** MVR and MFR of white, yellow, red, blue, and black flakes. Pictures of the flakes are also reported.

Flakes Color	Picture	MVR (cm^3^/10 min)	MFR (g/10 min)
WHITE	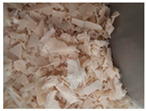	16.9 ± 2.0	14.6 ± 2.3
YELLOW	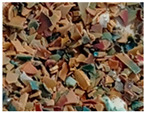	10.3 ± 1.8	8.8 ± 2.1
RED	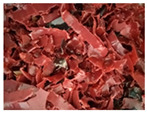	22.4 ± 3.9	18.9 ± 4.6
BLUE	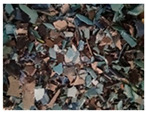	51.2 ± 6.0	41.7 ± 7.4
BLACK-1	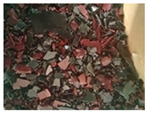	65.5 ± 4.7	55.1 ± 5.6
BLACK-2	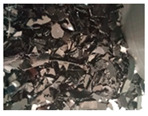	74.5 ± 5.8	70.5 ± 6.1

**Table 4:** MVR and MFR of rG-PET compounds.

Compound	MVR (cm^3^/10 min)	MFR (g/10 min)
DC	44.5 ± 2.5	47.5 ± 2.7
DC-An	38.2 ± 6.1	44.7 ± 7.1
DC-Ox	41.9 ± 3.7	46.5 ± 4.1
DC-J	26.1 ± 2.6	29.4 ± 2.9
LC	24.25 ± 0.7	27.7 ± 0.8
LC-An	9.9 ± 1.7	11.6 ± 1.9
LC-Ox	18.3 ± 2.7	21.0 ± 3.1
LC-J	4.8 ± 0.9	5.5 ± 1.0

**Table 5:** Tensile properties of rG-PET compounds.

Compound	Young’s Modulus (MPa)	Stress at Yield (MPa)	Elongation at Yield (%)	Elongation at Break (%)
DC	2461 ± 191	//	//	1.8 ± 0.5
DC-An	2730 ± 55	//	//	2.0 ± 0.3
DC-Ox	2363 ± 70	51.5 ± 1.8	3.2 ± 0.1	152 ± 95
DC-J	2489 ± 186	59.4 ± 9.1	3.6 ± 0.2	35.0 ± 12
LC	2545 ± 231	//	//	2.4 ± 0.4
LC-An	2281 ± 23.0	57.1 ± 2.3	3.8 ± 0.1	43 ± 21
LC-Ox	2406 ± 219	52.5 ± 8.7	3.2 ± 0.3	171 ± 12
LC-J	2280 ± 79	55.7 ± 2.6	3.7 ± 0.1	11 ± 6

**Table 6:** Flexural properties of rG-PET compounds.

Compound	Flexural Modulus (MPa)	Stress at Yield (MPa)	Stress at Break (%)	Deformation at Break (%)
DC	1951 ± 135	//	54 ± 17	3.4 ± 1.5
DC-An	1779 ± 42	//	47 ± 6	2.9 ± 0.4
DC-Ox	1886 ± 239	66 ± 10	59 ± 11	6.3 ± 2.5
DC-J	2230 ± 181	//	54 ± 9	4.6 ± 1.7
LC	2289 ± 106	//	46 ± 5	2.1 ± 0.2
LC-An	2280 ± 102	//	49 ± 8	2.3 ± 0.6
LC-Ox	2355 ± 411	80 ± 7	60 ± 23	5.9 ± 1.3
LC-J	2323 ± 164	//	59 ± 8	2.8 ± 0.6

**Table 7:** Shrinkage percentage for rG-PET compounds.

Compound	Flow Direction (%)	Transverse Direction (%)
DC	0.97	2.72
DC-An	0.98	2.73
DC-Ox	0.96	2.81
DC-J	0.85	2.73
LC	0.91	2.60
LC-An	0.91	2.84
LC-Ox	0.92	2.36
LC-J	0.79	2.28

**Table 8:** Results of the DSC characterization.

Compound	Tm (°C)	Tc (°C)	ΔHm (J/g)	Crystallinity (X) (%)
DC	252	201	49.8	35.6
DC-An	253	202	49.0	35.0
DC-Ox	250	200	54.2	39.0
DC-J	251	201	50.1	35.7
LC	256	206	45.1	32.2
LC-An	253	204	49.8	35.6
LC-Ox	254	204	46.7	33.4
LC-J	255	205	48.7	34.8

**Table 9:** Variation of MFR and ductility improvement observed for the chain-extended samples.

Compound	Maximum Number of Linkages per Molecule	ΔMFR (g/10 min)	Ductility Improvement % (Tensile)
DC-An	4	6.3	11
DC-Ox	2	2.6	8344
DC-J	23	18.4	1844
LC-An	4	14.35	1692
LC-Ox	2	5.95	7025
LC-J	23	19.45	358

**Table 10:** Summary of how each chain extender affects different properties.

Chain Extender	Macromolecular Design	Melt Fluidity (g/10 min)	Ductility (Elong. %)
Ox	Linear	High (>20)	High (>150)
An	Slightly branched	Medium (>10)	Medium
J	Branched	Low (>5)	Medium

## Data Availability

Dataset available on request from the authors.
